# Comparative Study on the Antimicrobial Activities and Metabolic Profiles of Five *Usnea* Species from the Philippines

**DOI:** 10.3390/jof9111117

**Published:** 2023-11-17

**Authors:** Thomas Edison E. dela Cruz, Lawrence P. Timbreza, Ek Sangvichien, Kin Israel R. Notarte, Krystle Angelique A. Santiago

**Affiliations:** 1 The Graduate School, University of Santo Tomas, España Blvd., Manila 1015, Philippines; lawrence.timbreza@gmail.com; 2 Fungal Biodiversity, Ecogenomics and Systematics-Metabolomics (FBeS) Group, Research Center for the Natural and Applied Sciences, University of Santo Tomas, España Blvd., Manila 1015, Philippines; 3 Department of Biological Sciences, College of Science, University of Santo Tomas, España Blvd., Manila 1015, Philippines; 4 Department of Biology, Faculty of Science, Ramkhamhaeng University, Hua Mark Bangkapi, Bangkok 10240, Thailand; eks@ru.ac.th; 5 School of Science, Monash University Malaysia, Jalan Lagoon Selatan, Bandar Sunway 47500, Malaysia; krystle.santiago1@monash.edu

**Keywords:** bioactivities, ESKAPE pathogens, fruticose lichens, lichen acids, secondary metabolites

## Abstract

The rapid emergence of resistant bacteria is occurring worldwide, endangering the efficacy of antibiotics. Hence, there is a need to search for new sources of antibiotics that either exhibit novel structures or express a new mechanism of action. The lichen *Usnea*, with its wide range of unique, biologically potent secondary metabolites, may solve this problem. In this study, *Usnea* species were collected in the Northern Philippines, identified through combined morphological and biochemical characterization, and tested for antimicrobial activities against the multidrug-resistant ESKAPE pathogens, i.e., *Enterococcus faecalis*, *Staphylococcus aureus*, *Klebsiella pneumoniae*, *Acinetobacter baumannii*, *Pseudomonas aeruginosa*, and *Enterobacter cloacae*, two standard antibiotic-sensitive test bacteria, and a yeast. A total of 46 lichen specimens were collected and later identified as *Usnea baileyi* (10), *U. diffracta* (10), *U. glabrata* (12), *U. longissima* (4), and *U. rubicunda* (10). The results show that the crude extracts of the *Usnea* species exhibited promising in vitro inhibitory activities against standard antibiotic-sensitive (*E. faecalis* ATCC 29212) and multidrug-resistant (methicillin-resistant *S. aureus* and *E. faecalis*) Gram-positive bacteria. Additionally, lichen compounds of representative specimens per species were identified and profiled using thin-layer chromatography (TLC) and high-performance liquid chromatography (HPLC). The detection of lichen acids (LA) via HPLC showed the presence of 24 peaks of lichen acids. TLC-bioautography identified the bioactive lichen acids as alectronic acid, connorstictic acid, consalazinic acid, diffractaic acid, echinocarpic acid, erythrin acid, galbinic acid, hypoconstictic acid, hyposalazinic acid, hypostictic acid, lobaric acid, menegazzaic acid, micareic acid, pannarin, salazinic acid, stictic acid, and usnic acid. Our study highlighted the wide spectrum of opportunities for using lichens for the discovery of potential antimicrobial agents.

## 1. Introduction

Natural products have played a significant role in the development of novel drugs as a rich source of various novel biologically active compounds. Lichens represent one of the more promising potential reservoirs of biologically active molecules, with well-documented biological activities such as antiviral [1], antibiotic [2], anti-inflammatory [3], anti-mycobacterial [4], antitumor [5], and other bioactivities.

Lichens are considered as a mutualistic relationship between a fungus (the mycobiont), mostly belonging to the phylum Ascomycota, and an alga or a cyanobacterium (the photobiont or photosynthetic partner), forming a complex and intimate biological union [6]. The biological activities exhibited by lichens are attributed to the secondary metabolites possessed by these organisms, which are mostly produced by the fungal symbionts and secreted onto the thallus (vegetative body) either in liquid or crystal forms [7]. Usually, these crystals constitute depsides and depsidones [8], pulvinates [9], and dibenzofurans [10]. The green algal and cyanobacterial symbiont also produced secondary metabolites in quantities smaller than that of the fungus [8]. Although these lichen substances are synthesized by the fungal partner, the interaction between the two symbionts is a crucial part of the synthesis of these lichen substances, as individual fungal components isolated from lichens do not always produce these metabolites in vitro [11], although the media component could influence the enhanced production of secondary metabolites [8]. Lichen substances often constitute 20% of the dried thallus, but in some forms, they account for 5 to 10% of the total dried weight [11]. Previous studies showed that the fruticose lichen *Usnea* [12,13,14,15,16,17], including those found in the Philippines [18,19,20], are potential agents against Gram-positive bacteria, thus making *Usnea* a promising source of bioactive secondary metabolites against multidrug-resistant Gram-positive bacterial pathogens such as MRSA (methicillin-resistant *Staphylococcus aureus*), VRSA (vancomycin-resistant *Staphylococcus aureus*), and VRE (vancomycin-resistant Enterococci), to name a few. A few other fungal associates of tropical *Usnea* species have been shown to be capable of producing bioactive secondary metabolites [21,22] and therefore are also promising organisms for drug discovery research [23].

The lichen *Usnea* was first described by Dillenius in 1742 [24]. It was described as a beard-like, lichenized ascomycete with a shrubby to pendent thallus that comprised string-like structures with radial symmetry and a cartilaginous central axis that can be uncovered by elongating the cortex [25,26]. The members of the genus *Usnea* are categorized under the Parmeliaceae family, with roughly 1000 extant species. They have a wide distribution, from tropical areas to polar zones [27]. The Philippines, despite having an advantageous climate for its growth, has conducted limited studies on the secondary metabolites and bioactivities of *Usnea*. An increase in the number of *Usnea* species has been reported in this country, from the early extensive reports of 32 species to an updated number of 81 species [28], and hence, this study examined and investigated the potential antimicrobial activities of Philippine *Usnea* species, particularly in targeting multidrug-resistant (MDR) bacteria.

## 2. Materials and Methods

### 2.1. Collection of the Fruticose Lichen Usnea

**Figure 1:**
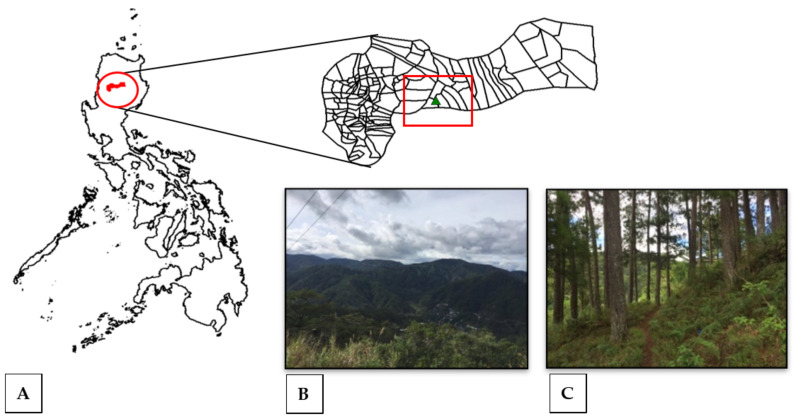
(**A**) The sampling localities—Mt. Amuyao, Mountain Province, with collection plots generated using DIVA-GIS ver. 7.4 and (**B**,**C**) photographs of forest sites where *Usnea* specimens were collected.

Specimens of the lichen *Usnea* were randomly collected along the trail leading to Mount Amuyao (17°2′15″ N, 121°6′4″ E) at the northeastern edge of the Cordillera region and the highest point at 2200 m above sea level (masl) in Mountain Province, Luzon Island, Northern Philippines (Figure 1). The study site has a Type I climate with relatively cool and humid conditions throughout the year, an ideal climatic condition for the growth of *Usnea*. The mountain is covered with thick forest usually dominated by *Pinus* spp. and *Dacarycarpus* spp. [29]. The collected specimens were manually cleaned by removing the soil debris and placed separately in paper bags. Herbarium materials of the collected specimens were prepared as voucher specimens and deposited at the Mycology Laboratory, Research Center for the Natural and Applied Sciences, University of Santo Tomas, in Manila, Philippines.

### 2.2. Characterization and Identification of Usnea

#### 2.2.1. Morphological Characterization

The collected lichen specimens were identified based on a comparison of morphometric data with published identification keys, e.g., Swinscow and Krog (1975), Clerc (1998), Herrera-Campos et al. (1998), McCune et al. (2005), Randlane et al. (2009), Ohmura et al. (2010), Ohmura (2012), Truong and Clerc (2012), and Shukla et al. (2014) [25,26,30,31,32,33,34,35,36]. Morphological characters such as thallus growth form, texture, color, length, description of branches and branchlets, the presence and absence of apothecium, and other reproductive structures were recorded and compared with the descriptions on these ID keys. Furthermore, microscopic images of the obtained specimens were also observed under standard compound light and stereomicroscopes. The identities of the lichen samples were also compared with herbarium specimens at the Lichen Research Unit in Ramkhamhaeng University, Thailand.

#### 2.2.2. Thalline Spot Test

The thalline spot test is considered as a standard procedure in lichen taxonomy. Although the use of spot tests, for example, the C+ reaction, does not permit the identification of specific metabolites, this remains useful in identifying species based on their medullary reactions [25,26,30,32,33,34,35,36]. The standard thalline spot test was conducted for the collected *Usnea* specimens. Three tests, namely the K test, C test, and KC test, were performed using two chemical reagents, potassium hydroxide (KOH) and sodium hypochlorite (NaOCl). For the K test, a drop of KOH was spotted on the exposed medulla of the lichen specimen. Similarly, a drop of NaOCl was spotted on the exposed medulla of the specimen for the C test. Finally, a drop of KOH and NaOCl was spotted simultaneously on another part of the exposed medulla of the specimen. Any change in color signified a positive result. All tests were carried out on different parts of the lichen thalli, i.e., the cortex, axis, and medulla. The color change in the cortex, medulla, and/or central cord was recorded in this study. The additional information gained from the results of these thalline spot tests was used to confirm the lichen identity.

### 2.3. Extraction of Lichen Metabolites

The extraction of lichen metabolites was performed as described by Santiago et al. [19]. Briefly, lichen specimens were air-dried for 24 h. After air drying, the lichen thalli were cut into small pieces and pulverized using a mortar and pestle to yield approximately one gram (g) of the powdered lichen specimen. Then, the powdered specimens were transferred into a screw-capped test tube (Pyrex, Châteauroux, France, 150 mm × 16 mm) and soaked with 10 mL of laboratory-grade acetone for 24 h. After soaking, the lichen extracts were filtered using a funnel and filter paper and stored in pre-weighed vials. The lichen crude extracts were concentrated via air-drying until the solvent was fully evaporated. The crystal-like lichen acids were weighed, and the percentage yield was calculated using the formula below:

$$\%\;\mathrm{yield}\;\mathrm{extract}=\frac{WCE}{WLT}$$

where *WCE* is the weight of the crude extract and *WLT* is the weight of the lichen thalli.

To reconstitute the lichen acids, acetone was added to each bottle containing the lichen substances to reach a final concentration of 10 mg/mL. The reconstituted lichen extracts were filter-sterilized through a 0.45 μm membrane filter and stored in a refrigerator until further use.

### 2.4. Determination of Antimicrobial Activities Using Paper Disk Diffusion Assay

#### 2.4.1. Test Bacteria

The multidrug-resistant strains of ESKAPE pathogens (*Enterococcus faecalis*, *Staphylococcus aureus*, *Klebsiella pneumoniae*, *Acinetobacter baumannii*, *Pseudomonas aeruginosa*, and *Enterobacter cloacae*) and two standard antibiotic-sensitive test bacteria (*E. faecalis* ATCC 29212 and *S. aureus* ATCC 25923) were obtained from the Research Institute for Tropical Medicine (RITM) in Muntinlupa City, Metro Manila, Philippines. The drug resistance profiling of these bacterial strains was previously conducted by RITM following the Clinical and Laboratory Standard Institute (CLSI) Antimicrobial Susceptibility Testing (AST) standards. All test bacteria were cultured on Blood Agar plates (BAP, Becton Dickinson, Franklin Lakes, NJ, USA).

#### 2.4.2. Preparation of Bacterial Inoculum

Each bacterium was first streaked on a BAP and incubated at 37 °C for 18–24 h. After incubation, each 24 h old bacterium was suspended in sterile Normal Saline Solution (NSS), and the turbidity was adjusted to that of 0.5 McFarland standard using a densitometer.

#### 2.4.3. Positive and Negative Controls

The antibacterial drugs Ampicillin (Oxoid, Waltham, MA, USA, 10 μg/mL), Doripenem (Oxoid, 10 μg/mL), and Sulfamethoxazole (25 μg/mL) were used as positive controls in the paper disk diffusion assay. Acetone was used as a negative control.

#### 2.4.4. Paper Disk Diffusion Assay

The lichen crude extracts were tested against the standardized ESKAPE bacterial pathogens and two standard antibiotic-sensitive test bacteria, *E. faecalis* ATCC 29212 and *S. aureus* ATCC 25923. The standardized test bacteria were initially swabbed on solidified Mueller–Hinton Agar (MHA, Becton Dickinson, Franklin Lakes, NJ, USA) plates (~4 mm deep). Then, 20 μL of the lichen crude extracts was added onto sterile paper disks and air-dried for approximately 10–15 s or until the solvent evaporated, giving a final concentration of 200 μg per disk of extract. These disks (Whatmann, Maidstone, Kent, UK, 6 mm, diam.) were carefully placed on top of the culture agar swabbed with the test bacteria. The positive and negative controls were similarly placed together with the test lichen extracts. The test was conducted in triplicate for each extract. All culture plates were incubated at 37 °C for 18–24 h. Following the incubation period, the zones of inhibition (ZOIs) were measured using a Vernier caliper (in mm), as similarly described in Notarte et al. [37].

### 2.5. Determination of Antimicrobial Activities Using TLC-Bioautography

#### 2.5.1. Test Bacteria

The standard antibiotic-sensitive strains of *E. coli* ATCC 25922 and *S. aureus* ATCC 25923 and the yeast *Candida albicans* ATCC 90028 were obtained from the Lichen Research Unit in Ramkhamhaeng University, Thailand. The standard antibiotic-sensitive test bacteria were maintained on Nutrient Agar (NA, Beckton Dickinson, Franklin Lakes, NJ, USA) while the yeast was maintained on Saboraud Dextrose Agar (SDA, Beckton Dickinson, Franklin Lakes, NJ, USA). The multidrug-resistant (MDR) bacteria *E. faecalis* and *S. aureus* were also included in this assay.

#### 2.5.2. Preparation of Inoculum and TLC Plates

Bacterial and yeast suspensions were prepared from 24 h old cultures and adjusted to the 0.5 McFarland standard. For the preparation of the TLC plates, the lichen crude extracts were initially spotted on a silica gel TLC plate. The TLC plate was then run in solvent system A (36:9:1 toluene/dioxane/glacial acetic acid, *v*/*v*/*v*). The TLC plate was air-dried for approximately 30 min to eliminate any traces of the solvents and the lichen spots were visualized under UV light at 245 nm and 350 nm wavelengths [18].

#### 2.5.3. Thin-Layer Chromatography—Bioautography

To determine the identity of bioactive lichen acids, 40 µL of the bacterial suspension was mixed with 40 mL of cooled and semi-solid MHA and was labeled as the seeded layer. A base medium, approximately 60 mL of solidified MHA, was also prepared and poured onto a sterile, disposable Petri plate. Once the base medium solidified, the TLC plate was placed on top of the agar (with the silica side of the TLC plate facing upwards) using sterile forceps. Then, the seeded MHA was carefully poured over the TLC plate. All culture plates were refrigerated for two hours for the direct diffusion of lichen acids without allowing the test organism to grow [18]. Culture plates were then incubated at 37 °C for 18–24 h. After the incubation period, spots of clearing zones and zones of inhibition were observed. The identity of the bioactive lichen compounds was determined by comparing them with the separately visualized TLC plates.

### 2.6. Chromatographic Analysis for Lichen Acid Profiling

#### 2.6.1. Thin-Layer Chromatography

Crude lichen extracts and the lichen acid standards, atranorin, norstictic acid, and salazinic acid, were initially spotted on TLC plates (Silica Gel 60 F_254_ aluminum plates, Sigma-Aldrich, St. Louis, MO, USA). The plates were run in two solvent systems, solvent system A—36:9:1 toluene/dioxane/glacial acetic acid (*v*/*v*/*v*)—and solvent system G—139:83:8 toluene/ethyl acetate/formic acid [38]—and then each TLC plate was sprayed with 10% sulfuric acid and heated using TLC Plate Heater III (Camag, Mutend, Switzerland) at 110 °C for 10 min. Similarly, spots were also observed by exposing the TLC plates to ultraviolet light at 245 nm and 350 nm wavelengths. Identification of the lichen spots was carried out following a comparison with the lichen acid standards.

#### 2.6.2. High-Performance Liquid Chromatography

Metabolic profiling of the five species of *Usnea* was performed following the protocol of Nguyen et al. [39]. Initially, the acetone extracts of the five *Usnea* species were re-dissolved in acetone to a final concentration of 10 mg/mL. The lichen extracts were subsequently subjected to HPLC (Shimadzu, Kyoto, Japan, LC-20A) with the following materials: YMC-Pac ODS-A (15,063.9 mm I.D.) reversed-phase column fully end-capped C18 materials (particle size, 5 mm; pore size, 12 nm). The column conditions included a temperature of 40 °C, a solvent system consisting of methanol/water/phosphoric acid (80:20:1), and an elution process at a flow rate of 1 mL/min. Using a photodiode array detector (SPD-M20A, Shimadzu, Kyoto, Japan), peaks were detected between 190 and 400 nm. An amount of 1 mL of the lichen extracts was used for HPLC in this study.

## 3. Results

### 3.1. The Collected Usnea Species

A total of 46 specimens were collected in Mount Amuyao, Mountain Province, in Northern Luzon, the Philippines. Based on morphoanatomical analysis (Table S1) and the results of the thalline spot tests (Table 1), the 46 lichen specimens were identified as belonging to five species of *Usnea* and distributed as follows: *U. baileyi* (10), *U. diffracta* (10), *U. glabrata* (12), *U. longissima* (4), and *U. rubicunda* (10).

### 3.2. Metabolic Profiles of Usnea Species

A total of 26 lichen acids were detected on the five *Usnea* species using solvent systems A and G (Table 2). As expected, differences in lichen acids were detected using the two solvent systems: 20 lichen acids with solvent system A and 17 lichen acids with solvent system G. Nine were specifically detected using solvent system A, while six were unique to solvent system G. However, eleven lichen acids were detected in both solvent systems used.

**Figure 2:**
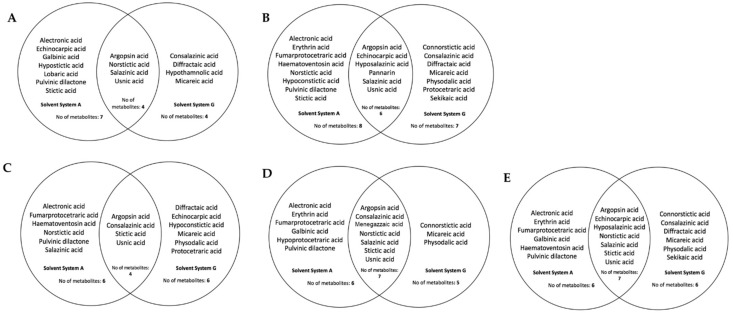
Lichen acids detected in *U. baileyi* (**A**), *U. diffracta* (**B**), *U. glabrata* (**C**), U. longissimima (**D**), and *U. rubicunda* (**E**) based on TLC using solvent systems A and G.

We also looked at and compared the metabolic profiles of each *Usnea* species reported in this study. The TLC profiles of *U. baileyi* showed 15 lichen acids (Figure 2A), with alectronic acid, echinocarpic acid, galbinic acid, hypostictic acid, lobaric acid, pulvinic dilactone, and stictic acid being detected only with solvent system A, whereas consalazinic acid, diffractaic acid, hypothamnolic acid, and micareic acid were unique to solvent system G. *U. diffracta* had 21 lichen acids (Figure 2B). Alectronic acid, erythrin acid, fumarprotocertraric acid, haematoventosin acid, norstictic acid, hypoconstictic acid, pulvinic dilactone, and stictic acid were detected with solvent system A, whereas connorstictic acid, consalazinic acid, diffractaic acid, micareic acid, physodalic acid, protocetraric acid, and sekikaic acid were detected with solvent system G.

The lichen extracts of *U. glabrata* had 16 lichen acids (Figure 2C). Solvent system A identified alectronic acid, fumarprotocetraric acid, haematoventosin acid, norstictic acid, pulvinic dilactone, and salazinic acid, while solvent system G identified diffractaic acid, echinocarpic acid, hypoconstictic acid, micareic acid, physodalic acid, and protocetraric acid. On the other hand, *U. longissima* had 18 lichen acids (Figure 2D). Alectronic acid, erythrin acid, fumarprotocetraric acid, galbinic acid, hypoprotocetraric acid, and pulvinic dilactone were recorded with solvent system A. A smaller number of lichen acids, i.e., only connorstictic acid, micareic acid, and physodalic acid, were detected with solvent system G. Finally, the lichen extracts of *U. rubicunda* were determined to have 19 lichen acids using solvent systems A and G (Figure 2E). Similarly, alectronic acid, erythrin acid, fumarprotocetraric acid, galbininc acid, haematoventosin acid, and pulvinic dilactone were detected with solvent system A, while connorstictic acid, consalazinic acid, diffractaic acid, micareic acid, physodalic acid, and sekikaic acid were detected with solvent system G. The number and types of lichen acids as detected with the two solvents for each of the *Usnea* species are also illustrated in Figure 2. The TLC chromatograms of the lichen acids detected with both solvent systems are also shown in Figure S1.

In this study, we also evaluated whether extraction time/storage affects the number of metabolites that can be detected via TLC. Our results show that the freshly extracted lichen specimens (21 lichen acids) had a more diverse array and higher number of lichen acids than old/stored lichen specimens (12 lichen acids) (Figure S1).

**Figure 3:**
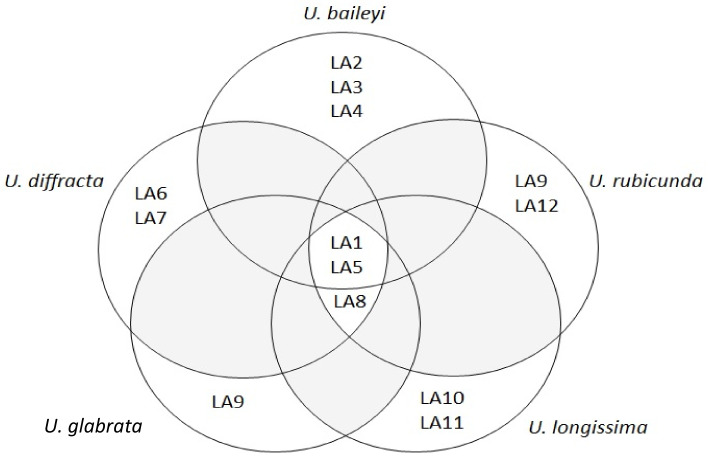
Venn diagram showing the similarities between lichen acids detected in the five species of *Usnea* based on their HPLC chromatograms.

The presence of lichen acids that are common and unique to a particular species of *Usnea* was also observed with HPLC (Figure S2). Two LAs were found in all lichen extracts. The crude extract of *U. baileyi* had unique lichen acids (LA2, LA3, LA4), while LA6 and LA7 were unique to *U. diffracta*. Only one lichen acid, LA9, was observed in the extracts of *U. glabrata*. LA10 and LA11 were unique to *U. longissima*, while LA9 and LA 12 were distinctive to *U. rubicunda*. Some of the observed peaks in the chromatogram were shared by three or more species of *Usnea*. A Venn diagram was created to show the shared lichen acids between the five *Usnea* species as determined using the HPLC chromatogram (Figure 3).

### 3.3. Antimicrobial Activities of Usnea

#### 3.3.1. Against Gram-Positive Antibiotic-Sensitive Test Bacteria

**Figure 4:**
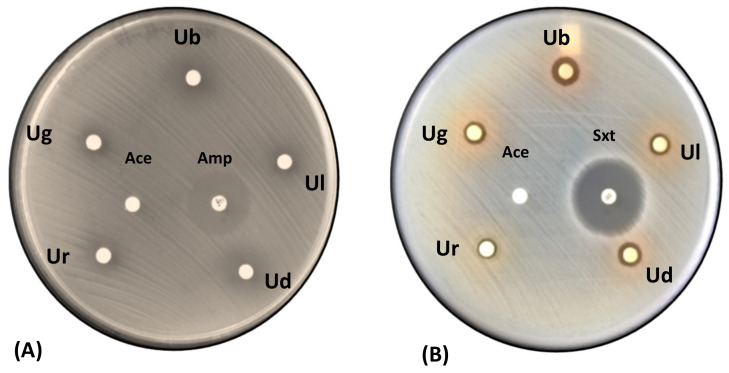
Zones of inhibition (ZOIs) exhibited by the lichen extracts of (Ub) *U. baileyi*, (Ud) *U. diffracta*, (Ug) *U. glabrata*, (Ul) *U. longissima*, and (Ur) *U. rubicunda* against (**A**) *E. faecalis* ATCC 29212 and (**B**) *S. aureus* ATCC 25923 in comparison with Ampicillin (Amp) and Sulfamethoxazole (Sxt). Acetone (Ace) served as the negative control.

In this study, the five lichen crude extracts showed inhibitory activities against the standard Gram-positive test bacteria based on the paper disk diffusion assay (Table 3, Figure 4). The results show that the lichen crude extracts exhibited weaker antibacterial activities against *S. aureus* ATCC 25923, whereas moderate (partially active) antibacterial activity was observed towards *E. faecalis* ATCC 29212.

#### 3.3.2. Against Multidrug-Resistant ESKAPE Bacterial Pathogens

**Figure 5:**
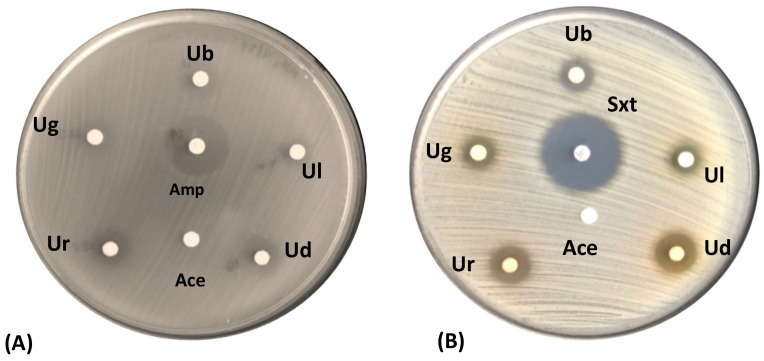
Zones of inhibition (ZOIs) exhibited by the lichen extracts of (Ub) *U. baileyi*, (Ud) *U. diffracta*, (Ug) *U. glabrata*, (Ul) *U. longissima*, and (Ur) *U. rubicunda* against multidrug-resistant strains of (**A**) *E. faecalis* and (**B**) *S. aureus* in comparison with Ampicillin (Amp) and Sulfamethoxazole (Sxt). Acetone (Ace) served as a negative control.

The resistance of disease-causing agents to readily available treatment options has raised concern for global health. These incidents are driven by a group of nosocomial pathogens that are acronymically dubbed as the “**ESKAPE**” bacterial pathogens—***E**nterococcus faecalis*, ***S****taphylococcus aureus*, ***K****lebsiella pneumoniae*, ***A****cinetobacter baumannii*, ***P****seudomonas aeruginosa*, and ***E****nterobacter cloacae*. To find a possible solution to the increasing prevalence of multidrug-resistant bacteria, we also tested our lichen crude extracts against MDR-ESKAPE pathogens. Our results show all of the tested lichen crude extracts exhibiting antibacterial activities against MDR strains of *S. aureus* (MRSA) and *E. faecalis* (Table 3, Figure 5) but not *K. pneumoniae*, *A. baumannii*, *P. aeruginosa*, or *E. cloacae*. The highest activity was found for the crude extracts of *U. diffracta*, with a ZOI of 14 mm.

### 3.4. Detection of Bioactive Lichen Acids Using TLC-Bioautography

**Figure 6:**
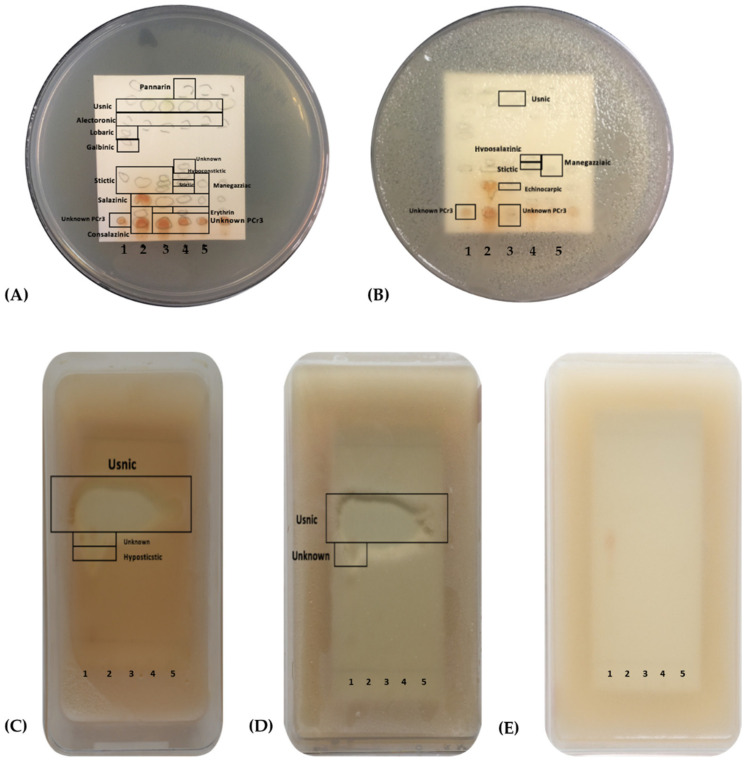
Bioactive lichen acids of (1) *U. baileyi*, (2) *U. glabrata*, (3) *U. rubicunda*, (4) *U. diffracta*, and (5) *U. longissima* against (**A**) *E. faecalis* and (**B**) methicillin-resistant *S. aureus*, (**C**) *E. coli* ATCC 25922, (**D**) *S. aureus* ATCC 25923, and (**E**) *C. albicans* ATCC 90028.

Along with 5 unknown bioactive lichen acids, a total of 14 bioactive lichen acids were detected through TLC-bioautography: alectronic acid, consalazinic acid, echinocarpic acid, erythrin acid, galbinic acid, hypoconstictic acid, hyposalazinic acid, hypostictic acid, lobaric acid, menegazzaic acid, pannarin, salanizic acid, stictic acid, and usnic acid. As observed in the TLC-bioautography, usnic acid consistently inhibited the test bacteria (Figure 6). In addition, unique bioactive lichen acids were detected in every species. Hypostictic acid and lobaric acid from *U. baileyi* were bioactive against the test bacteria. Pannarin from *U. diffracta* and menegazzaic acid from *U. longissima* were also bioactive, while hypoconstictic acid, which was common to both *U. diffracta* and *U. glabrata*, was bioactive against *E. faecalis*. Hyposalazinic acid, which was common to *U. diffracta* and *U. rubicunda*, was also bioactive against MRSA. We also detected bioactive metabolites that were shared by three or more species. Galbinic acid detected in *U. baileyi*, *U. longissima*, and *U. rubicunda*, and erythrin acid in *U. diffracta*, *U. longissima*, and *U. rubicunda* were bioactive. Echinocarpic acid, which was common to *U. baileyi*, *U. diffracta*, *U. glabrata*, and *U. rubicunda*, was bioactive against MRSA. Alectronic, consalazinic, salazinic, stictic, and usnic acids, which were identified in all five species of *Usnea*, were bioactive against the test bacteria. No activity was observed against yeast.

## 4. Discussion

Since overcoming the current challenge faced by pharmaceutical industries depends on the discovery of new sources of drugs, lichens are undeniably one of the best options. It has been established that lichens produce a plethora of bioactive secondary metabolites. In fact, lichen-acquired products and their antibacterial properties are of special interest to scientists, since half of all known lichens have been described to exhibit inhibitory activities against pathogenic microorganisms [40]. Among these promising lichens is the pendulous fruticose lichen *Usnea*. Despite the fact that the Philippines is home to a vast variety of lichen species, the antibiotic properties of the lichen *Usnea* remain largely understudied. In this study, the lichen *Usnea* was collected from Mount Amuyao, Northen Philippines, and tested for its bioactivities to find potential sources of pharmacologically valuable chemicals. This study therefore contributed to the growing list of the beneficial properties of Philippine lichens, which includes antioxidant [41], antibacterial [42], and herbicidal [43] activities. It has been known for many decades that lichens can be used in the pharmaceutical industry [15].

*Usnea* species contain a wide array of bioactive secondary metabolites [18,19,20]. For instance, *U. rubicunda* possesses stictic acid as its main substance, and norstictic and psoromic acids as accessories [34]. *U. glabrata* has barbatic acid as its main substance, and salazinic and usnic acids as accessories [34]. *U. diffracta* has diffractaic acid and usnic acid as its key substances, but barbatic acid may constitute 20% of its metabolites [20,26]. In this study, we detected 26 lichen acids from the five species of *Usnea*, with *U. diffracta* producing the highest number (21 lichen acids) followed by *U. rubicunda* (19), *U. longissima* (18), *U. glabrata* (16), and *U. baileyi* (15). Differences in the metabolic profiles of this same species may occur due to the extensive morphological plasticity exhibited by the genus *Usnea* [36]. For instance, the medullary chemistry of *U. fragilescens* was found to comprise usnic, norstictic, salazinic, and protocetraric acids [26]. However, in the subsequent years, Ohmura et al. [35] found that *U. fragilescens* exhibited another chemotype, which only included usnic, norstictic, and stictic acids. We also observed differences in metabolites due to specimen storage, as freshly extracted lichen thalli have a higher number of lichen acids and a higher diversity of acids than extracts of stored lichen thalli.

Thin-layer chromatography has greatly improved the rapid detection and recognition of lichen substances [38]. This technique is still widely used and readily available for identifying lichen acids routinely. In this study, lichen substances from the collected species of *Usnea* were determined and run on TLC using two solvent systems. As expected, the number and types of lichen acids differed between the two solvent systems, with solvent system A detecting more lichen acids than solvent system G. Interestingly, a high number of lichen acids, 11 to be exact, can be detected by both solvent systems. This finding supports the usual practice of using two solvent systems for the TLC profiling of lichen acids. Furthermore, the use of HPLC could improve the detection of lichen acids, particularly compounds that differ between species. Twelve lichen acids were recorded through HPLC, with only three compounds shared by at least three species. The lichen acids detected in Philippine *Usnea* were also found to differ from those reported in temperate countries. For example, the metabolite profile of *U. diffracta* consists of alectronic acid, erythrin acid, fumarprotocertraric acid, haematoventosin acid, norstictic acid, hypoconstictic acid, pulvinic dilactone, and stictic acid. On the other hand, Ohmura [26] reported that collected temperate specimens of *U. diffracta* from Taiwan showed the presence of baeomycesic, barbatic, diffractaic, squamatic, and usnic acids. Perhaps geographic origin viz-a-viz its climatic conditions could influence the production of lichen acids, but a detailed, comparative study with specimens of similar *Usnea* species collected from different ecoregions, for instance, between tropical and temperate countries or the Paleo- and Neotropics, is needed.

The differences in the lichen acid profiles observed in this study may also suggest differences in the fungal component or mycobiont, total loss of major compounds due to the dynamic interaction of numerous environmental factors (i.e., elevations, temperature fluctuations, and relative humidity), culture conditions, and the involvement of different secondary products with different biosynthetic pathways [44,45]. For instance, the production of lichen acids in the lichen *Umbilicaria americana* decreases as the elevation gradually increases, and the positive effects on the growth and production of usnic acid are due to nutritional factors [45,46]. It should also be noted that time has a crucial effect on the diversity of lichen acids, as observed in the TLC profiles of freshly extracted and stored lichen specimens in this study. Therefore, we suggest, as future research, looking into different factors that may contribute to the production of diverse lichen acids. Understanding the interplay between different abiotic/climatic and biotic factors in the production of lichen acids can aid researchers in designing optimized incubation conditions for the growth of and fermentation by lichens and/or their mycobionts.

Interestingly, we also recorded the presence of lichen acids that are unique to a particular species, particularly with the use of more sensitive detection equipment, such as HPLC. These lichen acids may be used to distinguish one species from another. Lichen chemistry, which is mainly based on the secondary metabolites found in lichens, was the most useful method for determining morphological characters before the advent of advanced molecular techniques [47]. As such, lichen acids were included as valuable criteria for the species delimitation of lichens, and have remained useful to date.

Over the past few decades, there has also been a growing interest in the study of lichens as possible sources of novel, pharmacologically active biomolecules [40]. These compounds can be used for chemotherapeutic therapies against cancer and other diseases. In fact, the structures of more than 1050 different lichen substances have already been reported [48]. Screening these metabolites can decode their biological properties, such as their antioxidant [41,49], cytotoxic [39], and antimicrobial [18,19,20] activities. The species of *Usnea* reported in this study exhibited antibacterial activities, particularly those targeting both antibiotic-sensitive and multidrug-resistant Gram-positive bacteria. The antibacterial properties of lichens can be attributed to the presence of active lichen substances [40]. For instance, depsides and depsidones are the most numerous classes of secondary metabolites in lichens and have been proven effective against various bacteria [15]. Moreover, other studies have provided evidence of lichens producing usnic acid that demonstrates higher inhibition against drug-sensitive bacteria [15,18,19,20,50,51,52]. For instance, Santos et al. [53] and Santiago et al. [18,19,22] examined the inhibitory activities of fruticose lichens collected from different islands of the Philippines. Their results showed that most of the lichen extracts were active against *Bacillus subtilis* and *S. aureus*. The aforementioned studies have demonstrated promising results against *S. aureus*, including the multidrug-resistant strains.

The involvement and synergy of different bioactive lichen acids contribute to the antibacterial properties of lichen crude extracts against Gram-positive MDR bacteria, as supported by the TLC-bioautography results in this study. Similar findings have been reported previously [9,54]. For instance, the lichen substances norstictic acid, salazinic acid, and usnic acid have been found to be effective against various pathogenic bacteria [11]. These compounds have the ability to act against microorganisms by interrupting the membrane function of bacterial cells [2]. It would be interesting now to look into different combinations of lichen acids and how these combinations affect bioactivities. On the other hand, none of the *Usnea* crude extracts exhibited antibacterial properties against Gram-negative MDR bacteria. As depicted in previous studies, most of the lichen extracts were more active against Gram-positive bacteria [18,19]. Recently, Timbreza et al. [20] also proved that most *Usnea* species are not active against Gram-negative MDR bacteria.

In summary, we identified five species of *Usnea* from a pine-dominated mountain forest in the Northern Philippines. We observed diversity in the bioactive metabolites produced by and extracted from the lichen thalli. Extraction of lichen acids from freshly collected lichen samples is an ideal method for obtaining more diverse compounds. We also reported the bioactivities exhibited by the lichen crude extracts against our test bacteria, particularly against drug-resistant Gram-positive bacteria. Our study highlighted the enormous potential of lichens for our efforts to develop new antibiotics to counter the rise of antimicrobial resistance.

## Figures and Tables

**Table 1:** Chemical reaction carried out in the cortex (C), axis (A), and medulla (M) as thalline spot test and percent yield of the five *Usnea* species.

Species	K Test ^a^			C Test			K + C Test			Percent Yield (WCE/WLT) ^b^
	C	A	M	C	A	M	C	A	M	
*U. baileyi*	Y/O	nr	nr	nr	nr	nr	PY/Y	nr	nr	4%
*U. diffracta*	nr	R	R	nr	nr	nr	nr	nr	nr	1%
*U. glabrata*	nr	Y/O	nr	nr	nr	nr	nr	PY/Y	nr	2%
*U. longissima*	nr	nr	nr	nr	nr	nr	P/Y	P/Y	nr	3%
*U. rubicunda*	nr	R	Y/O	nr	nr	nr	Y	O	nr	1%

^a^ Colors observed in the thalline spot test and their corresponding lichen acids: K test: Y (yellow) = atranorin, Y (yellow) to O (orange) = salazinic, Y (yellow) to B (brown) = protocetratic, R (red) to P (purple) = anthraquinones. C test: R (red) = m-dihydroxy phenols, G (green) = dihydroxy dibenzofurans. KC test: PY (pale yellow) to Y (yellow) = usnic acid, R (red) = depsides and depsidones; nr: no observable color change. ^b^ Percent yield is expressed as the weight of dried, crystalized crude extract (WCE) over the weight of the extracted lichen thalli (WLT).

**Table 2:** Lichen acids detected in the five *Usnea* species based on TLC profiling.

Solvent System	Lichen Acids	Total No. of Metabolites
A only	Alectronic acid Erythrin acid Fumarprotocetraric acid Galbinic acid Haematoventosin acid Hypoprotocetraric acid Hypostictic acid Lobaric acid Pulvinic dilactone	9
G only	Connorstictic acid Diffractaic acid Hypothamnolic acid Micareic acid Protocetraric acid Sekikaic acid	6
A and G	Argopsin Consalazinic acid Echinocarpic acid Hypoconstictic acid Hyposalazinic acid Menegazzaic acid Norstictic acid Pannarin Salazinic acid Stictic acid Usnic acid	11
Total Lichen Acids Detected:		26

Solvent system A: 36:9:1 toluene/dioxane/glacial acetic acid. Solvent system G: 139:83:8 toluene/ethyl acetate/formic acid.

**Table 3:** Zones of inhibition exhibited by the lichen crude extracts against the ESKAPE bacterial pathogens and two standard test bacteria.

	Test Bacteria Zone of Inhibition (mm) (200 μg/Disk)							
Lichen Extracts	Against Standard Bacteria		Against MDR Bacteria					
	*E. faecalis*	*S. aureus*	*E. faecalis*	MRSA	*K. pneumoniae*	*A. baumanii*	*P. aeruginosa*	*E. cloacae*
*U. baileyi*	14 ± 0.46	10 ± 0.58	10 ± 0.23	10 ± 0.58	0	*0*	*0*	*0*
*U. diffracta*	14 ± 1.16	10 ± 0.58	14 ± 0.98	14 ± 0.58	0	0	0	0
*U. glabrata*	10 ± 1.73	8 ± 1.55	9 ± 1.73	9 ± 1.15	0	0	0	0
*U. longissima*	12 ± 1.37	8 ± 1.33	9 ± 1.37	9 ± 1.33	0	0	0	0
*U. rubicunda*	13 ± 2.08	8 ± 0.29	13 ± 1.82	13 ± 0.29	0	0	0	0
Ampicillin	27 ± 0.46	-	23 ± 0.46	-	-	-	-	-
Sulfame- thoxazole	-	30 ± 0	-	28 ± 0	-	-	-	-
Doripenem	-	-	-	-	27 ± 0.58	28 ± 0.58	29 ± 0	27 ± 1.53
Acetone	0	0	0	0	0	0	0	0

## Data Availability

The data presented in the study are available on request from the corresponding authors.

## Supplementary Materials

The following supporting information can be downloaded at: https://www.mdpi.com/article/10.3390/jof9111117/s1, Table S1: key morphoanatomical characters of the collected Usnea species. Figure S1: TLC plates run in solvent system A (A,C) and solvent system G (B,D), showing the lichen metabolites detected in Usnea species. Lichen extracts were run on TLC immediately following extraction (A,B). Lichen extracts were stored at room temperature for 1 month before TLC (C,D). Figure S2: HPLC chromatogram of the lichen acids detected in the thallus of Usnea baileyi (A), Usnea diffracta (B), Usnea glabrata (C), Usnea longissima (D), and Usnea rubicunda (E).
