# Supplementary material for: Comparative Study on the Antimicrobial Activities and Metabolic Profiles of Five Usnea Species from the Philippines
Source: J Fungi (Basel). 2023 Nov 17;9(11):1117. doi: 10.3390/jof9111117 (PMC10672636; doi:10.3390/jof9111117)
Supplement: Supplementary file 1 [file jof-09-01117-s001.zip › jof-2661819-supplementary.pdf]

Table S1. Key morpho-anatomical characters of the collected *Usnea* species.

Taxa	No. of specimens	Image	Characteristics
U. baileyi	10	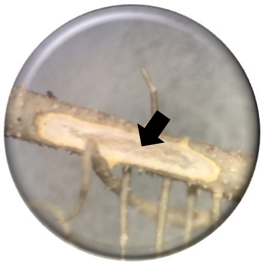	Erect to subpendent thallus with dark brown base, fistulose axis, punctiform soralia, and the presence of yellow subcortical pigmentation in the medulla near the cortex
U. diffracta	10	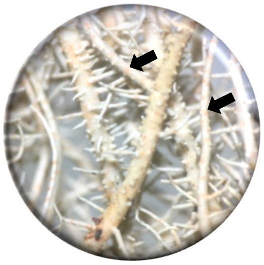	Erect to shrubby with anisotomic-dichotomous branching, presence of orange patches in the cortex, has a very stable cortex, annular-pseudocyphellae distinct on the segments of branches (bamboo-like appearance)
U. glabrata	12	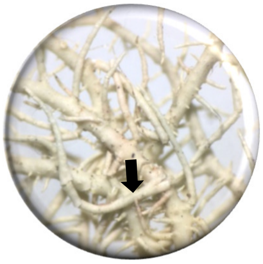	Small shrubby thallus with constriction of secondary branches at their base, presence of rather large soralia, and absence of both papillae and isidia, cortex thin and shiny with thick loose medulla and thin central axis
U. longissima	4	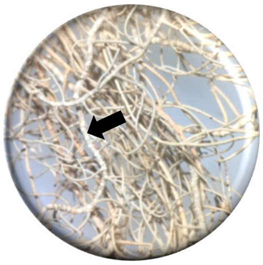	Pendent thallus with fish bone like appearance, presence of annular pseudocyphellae segment and terete and decorticated main branches
U. rubicunda	10	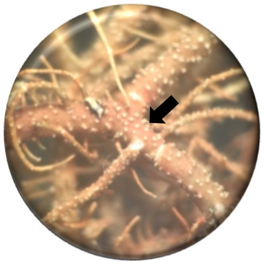	Erect to shrubby with anisotomic-dichotomous branching, non-elongated terminal and sub-terminal branches, presence of red pigment in the cortex, stipitate and rounded soralia, and absence of soralia on fibrils

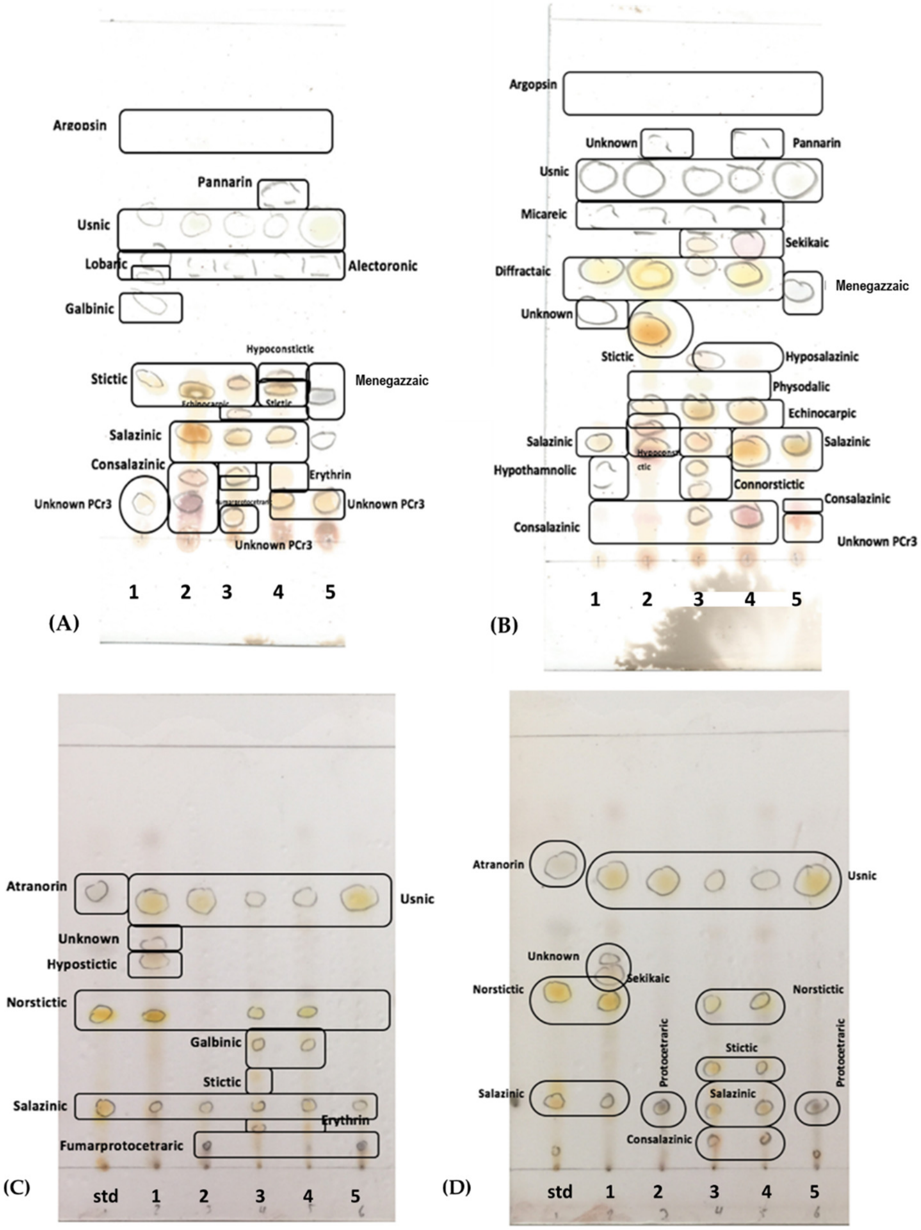

Figure S1. TLC plates run in solvent system A (A, C) and solvent system G (B, D) showing the lichen metabolites detected in *Usnea* species. Lichen extracts were run on the TLC immediately following extraction (A, B). Lichen extracts were stored at room temperature for 1 month before TLC (C, D).

A

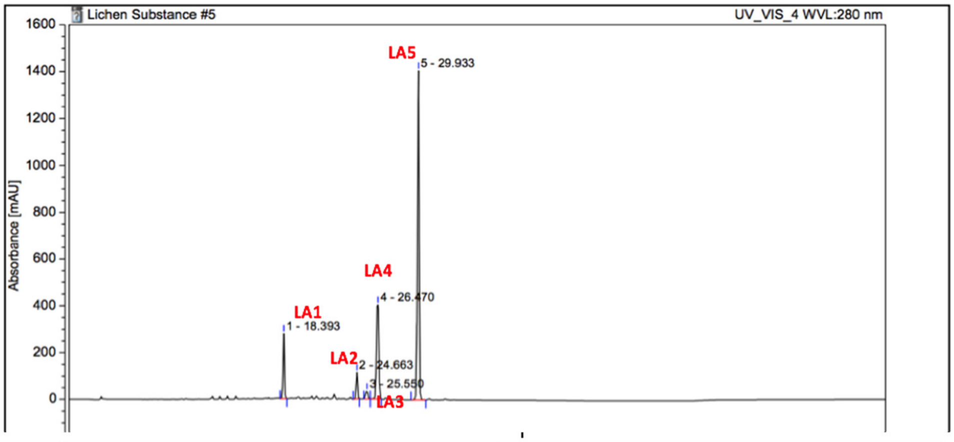

B

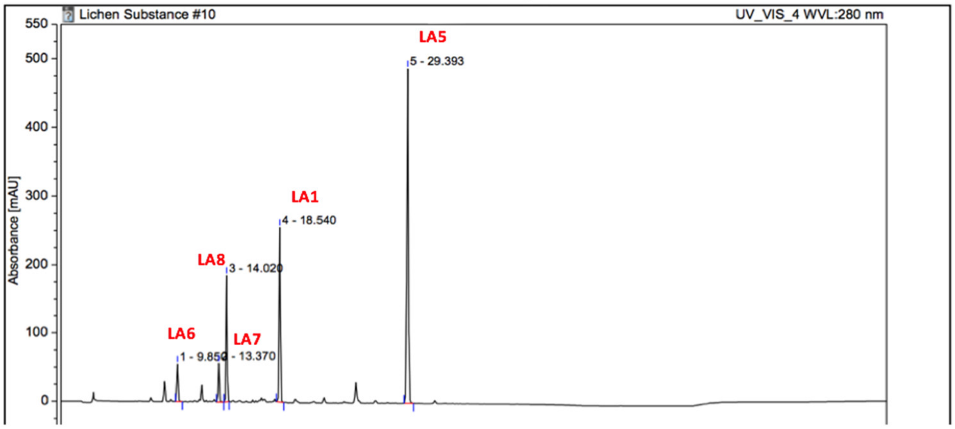

C

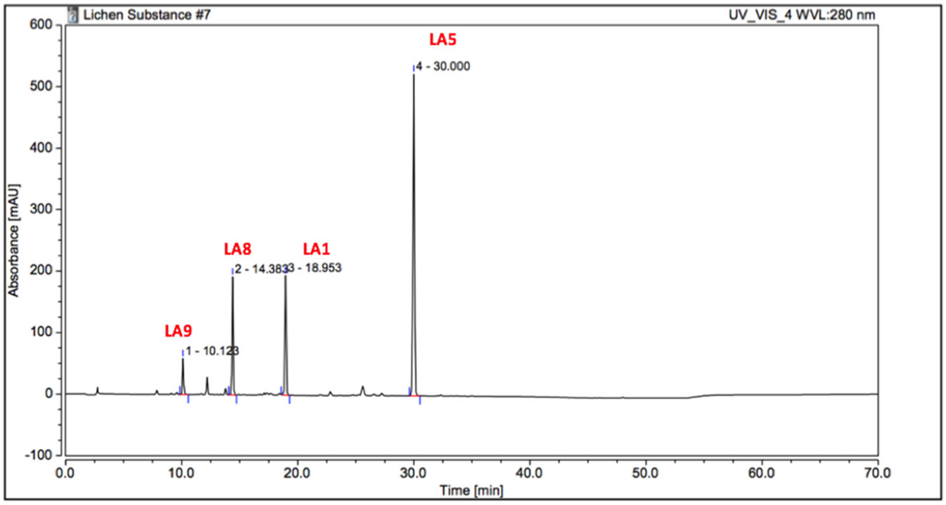

Figure S2. HPLC chromatogram of the lichen acids detected in the thallus of *Usnea baileyi* (A), *Usnea diffracta* (B), *Usnea glabrata* (C), *Usnea longissima* (D), and *Usnea rubicunda* (E).

D

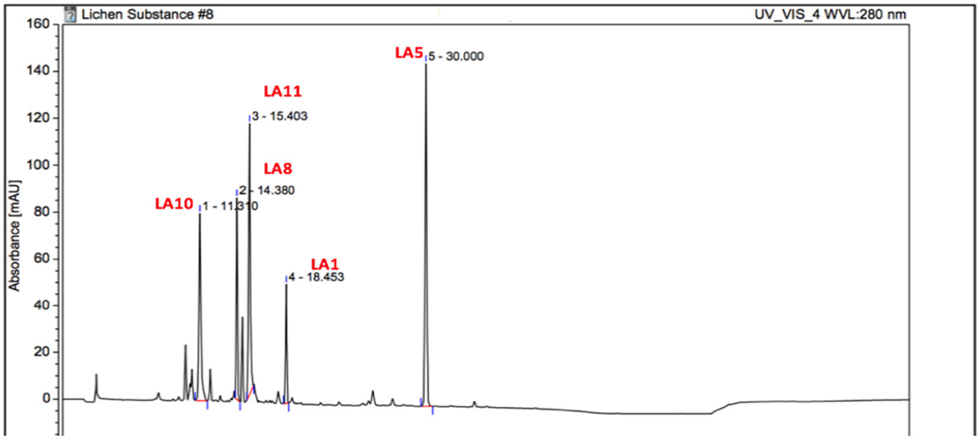

E

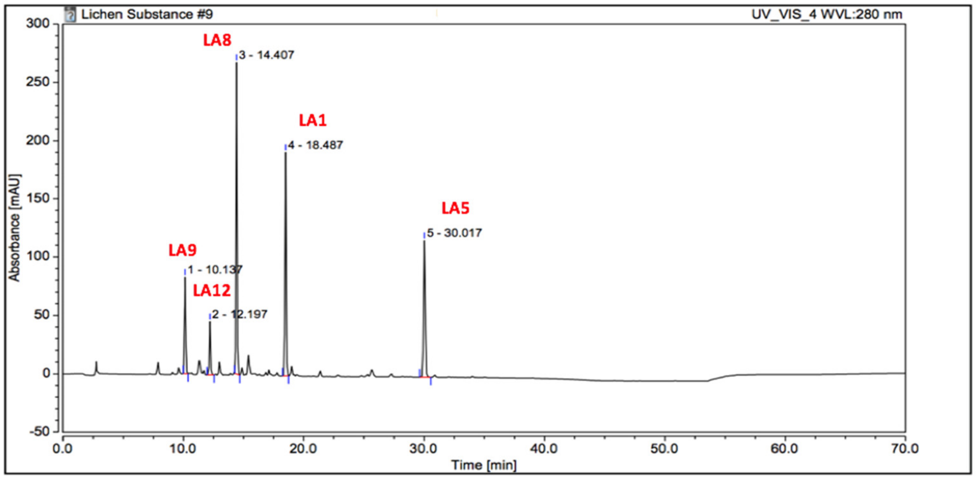

Figure S2. continuation.
